# Freiburg Neuropathology Case Conference: A 56-year-old Man Presenting with Progressive Gait Disorder, Neck Pain, and Lower Cranial Nerve Palsy

**DOI:** 10.1007/s00062-021-01114-1

**Published:** 2021-11-12

**Authors:** R. Sankowski, N. Lützen, U. Hubbe, M. Prinz, H. Urbach, D. Erny, C. A. Taschner

**Affiliations:** 1 Department of Neuropathology, University of Freiburg, Freiburg, Germany; 2 Department of Neuroradiology, University of Freiburg, Breisacherstraße 64, 79106 Freiburg, Germany; 3 Department of Neurosurgery, University of Freiburg, Freiburg, Germany; 4 Medical Centre, University of Freiburg, Freiburg, Germany

**Keywords:** IgG4-related hypertrophic spinal pachymeningitis, Spinal meningioma, Spinal lymphoma, Neurosarcoidosis, Hirayama disease

## Case Report

A 56-year-old man presented with deterioration of a pre-existing gait disorder and progressive neck pain. An autoimmune-mediated motoric axonopathy of the glossopharyngeal, hypoglossal and recurrent laryngeal nerves on the left side had been diagnosed 8 years previously and treated with intravascular immunoglobulins. In June 2020, a biopsy had been performed in view of progressive symptoms of cervical spinal stenosis with considerable thickening of the dura at the C3 level with an inconclusive result.

On clinical examination the patient presented with mild dysarthria, hypoglossal and glossopharyngeal paresis with tongue atrophy, deviation of the tongue to the left, and deviation of the uvula to the right. He showed a broad-based slightly ataxic gait pattern, the Romberg test was unstable, and Babinski reflexes were positive bilaterally. These findings were consistent with cervical myelopathy and basal cranial nerve dysfunction.

Electrophysiological testing revealed normal nerve conduction velocity of the sural nerves bilaterally, tibial SEP showed good lumbar potentials with normal latencies bilaterally, and cortical potentials showed borderline latencies on the right and normal latencies on the left. The cerebrospinal fluid showed a lymphocytic pleocytosis. Pathogen diagnostics including tuberculosis were negative.

We decided to take a new biopsy from the thickened cervical dura, which showed a strong uptake of contrast medium in order to obtain a definitive diagnosis.

The biopsy was planned at the level of C6 on the right side to avoid postoperative scar structures from the previous biopsy. A small caudal portion of the existing long-stretch skin scar was opened, the tissue was atraumatically dilated, and a 20-mm diameter tubular retractor was inserted down to the right hemilamina C6. Under microscopic view, the hemilamina C6 and the interlaminar window C6/7 were visualized. A diamond bur was used to perform a partial hemilaminectomy C6 and a rongeur was used to resect the ligamentum flavum. A tissue compatible with the dura was now revealed. This was cut through layer by layer with the diamond knife until cerebrospinal fluid emerged. The dura layer appeared thickened to 3–5 mm and had considerably hardened. A 5 × 10 mm block of the dura was resected and sent for histological work-up. Underneath, the myelon was clearly visible and CSF flowed freely. The severely thickened and indurated dura was not sufficiently mobilizable for primary closure. Therefore, the dura was closed by adhesion with Tachosil® (Takeda Pharmaceutical, Tokio, Japan) and a mixture of fibrin glue and Spongostan® (Ethicon, Somerville, NJ, USA). Thereafter, watertight conditions prevailed. Finally, retraction of the tubular retractor is performed with coagulation of microhemorrhage from the musculature, a subcutaneous suture and skin closure with glue. No drainage was applied. The patient did not present any new focal neurological deficit postoperatively and could be mobilized in a timely manner and was discharged on the third postoperative day.

## Imaging

**Fig. 1:**
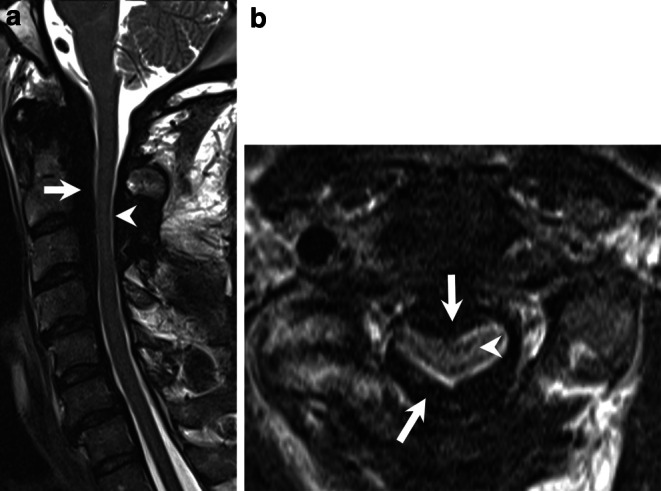
Sagittal (**a**) and axial (**b**, level C2/3) T2-weighted images showed a high-grade stenosis of the cervical canal between C2 and C4 (**a**, *arrowhead*). The stenosis was caused by a marked enlargement of the dura/epidural space extending from the foramen magnum down to C7. On T2-weighted images, the space-occupying lesion was hypointense (**a**, **b**, *arrows*). In addition, a centromedullary hypersignal corresponding to a cervical myelopathy was present (**b**, *arrowhead*)

**Fig. 2:**
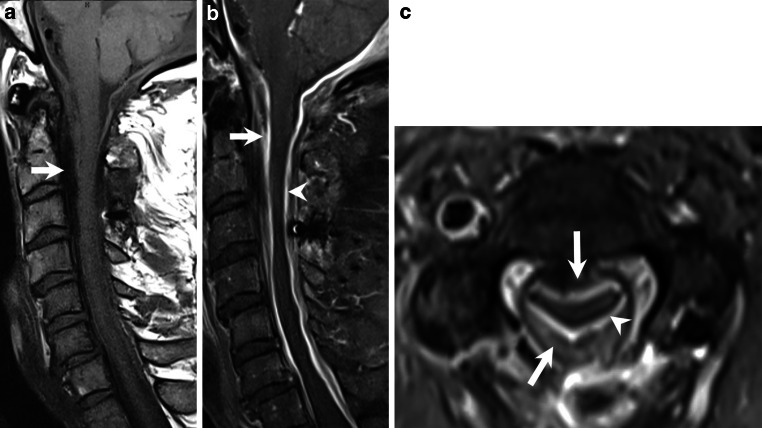
On sagittal native T1-weighted images (**a**) the space-occupying lesion also appears hypointense (*arrow*). On T1-weighted sagittal (**b**) and axial (**c**, level C2/C3) images after administration of gadolinium the meninges display homogeneous enhancement (**b**, **c**, *arrowhead*) whereas the space-occupying component shows an irregular enhancement pattern (**b**, **c**, *arrow*)

Magnetic resonance imaging (MRI) of the cervical spine revealed a high-grade stenosis of the cervical canal between C2 and C4. This stenosis was caused by a marked enlargement of the epidural space (Figs. 1 and 2) that extended from the foramen magnum down to the C7 level of the cervical spine. On T2-weighted images (Fig. 1a,b) and native T1-weighted images (Fig. 2a) the space-occupying lesion was hypointense. On T1-weighted images after administration of gadolinium (Fig. 2b,c) the meninges displayed homogeneous enhancement whereas the space-occupying component showed an irregular enhancement pattern. On computed tomography (CT) myelography the lesion had low densities, and no signs of osseous involvement were visible (not shown).

## Differential Diagnosis

### IgG4-related Hypertrophic Spinal Pachymeningitis

The entity previously known as idiopathic hypertrophic pachymeningitis (IHP) is a rare inflammatory disorder with unknown, most likely autoimmune origin. It mainly effects the intracranial pachymeninges. A second pathology, the IgG4-related sclerosing pachymeningitis (IgG4-RSP), a systemic lymphoproliferative fibrosis predominantly presenting with IgG4 infiltration shares many characteristic features with IHP. The IgG4-RSP belongs to the IgG4-related diseases (IgG4-RD) involving other organs such as the pancreas, salivary glands, retroperitoneum and many more [1]. There is evidence that both pathologies overlap or even have the same origin [2].

However, an affection of the cervical spinal cord is extremely rare in both entities and has been described in only a few case reports to date [3, 4]. There are no specific clinical symptoms until the proliferation leads to spinal compression with myelopathy and/or radiculopathy.

In MRI both entities show characteristic findings, which include an elongated dural mass with strong and homogeneous enhancement. On T2 weighted images IgG4-related Hypertrophic Spinal Pachymeningitis usually displays mild to marked hyperintense signals, with hypointense areas related to local fibrotic tissue [3, 5]. Pai et al. noted that the dural mass in IHP may be circumferentially or ventrally and/or dorsally localized. Involvement of the leptomeningeal structures or the arachnoidea have not been described [3]. MRI screening of other organs that are typically involved in a systemic IgG4-related disease can give further hints. Recurrences after treatment with steroids, immunosuppressants or even decompression surgery are common.

Considering the impressive T2 signal reduction in our patient and multiple relapses during the course of the disease, IHP or IgG4-RSP is a valid differential diagnosis.

### Spinal Meningioma

Spinal meningiomas are the second most common spinal tumors after spinal schwannomas. They are typically located on the internal face of the dura, predominantly at the thoracic level. Spinal meningiomas facing towards the epidural space are rare and account for 3.5–7% of all spinal meningiomas [6]. An exclusively “en plaque” configuration only occurs in 3.5% of all spinal extradural meningiomas [7]. It is characterized by infiltrative nature, sheet-like growth and occasionally extradural spread. Mean age of patients with epidural spinal meningiomas is 45 years with a clear female predominance [8]. Clinical symptoms basically depend on the degree of spinal cord compression.

On imaging spinal epidural meningiomas share the radiological characteristics of classical meningiomas with some specific differences. Spinal epidural meningiomas extend over 2–5 spinal segments and tend to be more aggressive (adherence to the dura, extension into intervertebral foramina, invasion in adjacent structures). They may appear hypointense in T2 weighted images more frequently due to calcification (57% vs. 1–4.6% in all spinal meningiomas) or fibrosis and they regularly exhibit a “dural-tail sign” (93% vs. 50–60% in intradural spinal meningioma) with a strong contrast enhancement [8]. Performing a CT scan to identify calcifications can be of major benefit for the diagnosis. Recurrence rate for epidural spinal meningioma is high because of the invasive character of the tumor.

Especially the “en plaque” variant of the spinal extradural meningioma encircles the spinal cord, forming a C-shape or even surrounds the spinal canal circumferentially [6, 8]. Given these characteristics in configuration and signal behavior in T2 and postcontrast images, it seems to be a valid differential diagnosis.

### Spinal Lymphoma

Spinal lymphomas are classified as paraspinal, vertebral and epidural depending on the site of manifestation, [9]. Epidural lymphomas account for 9% of all spinal epidural tumors; however, primary spinal epidural lymphomas are rare with a predominance for the thoracic spine. Clinical manifestation most commonly occurs in the fifth to seventh decade of life. Symptoms depend on mass affect to the spinal cord whereas a B-symptomatology can also be indicative of the disease.

MRI features are comparable to lymphoma manifestations in other locations. The tumor is isointense on T1 weighted images with marked homogeneous contrast enhancement. On T2 weighted images, signal intensities (SI) are isointense to hyperintense [9]. In rare cases, SI can also be hypointense [10]. Central nervous system (CNS) lymphomas typically show restricted diffusion in diffusion-weighted images (DWI) due to the hypercellularity of the tumor, making DWI helpful to determine the diagnosis of spinal epidural lymphomas [11]. Bony involvement may be seen on MRI or CT. In these cases, the tumor is most often located dorsally or laterally [9].

In particular, the prominent signal depression in the T2 weighted images makes the diagnosis of epidural lymphoma less likely.

### Hirayama Disease

Hirayama disease is a rare non-hereditary cervical myelopathy. The disease is characterized by asymmetric, slowly progressive atrophic muscle weakness. Underlying pathomechanism is a cervical compression resulting in microcirculatory changes related to anterior displacement of posterior cervical dura within anteflexion of the cervical spine. Etiology is still debated whereas one common theory postulates an insufficient growth of the dura during puberty leading to a pathologic laxity of the dura [12]. Hirayama disease is most frequent in Asia, whereas only single cases have been reported in Europa and North America. Finally, the incidence remains unclear [13]. Patients are mostly affected in the second and third decade of life with a female predominance.

Most characteristically on MRI is the progressive loss of attachment of the posterior dura comparing the neutral position to flexion leading to a compression of the spinal cord and widening of the posterior epidural space [13]; however even in neutral position there are imaging features like loss of dural attachment, asymmetric lower cervical cord atrophy and signs of myelopathy in axial images as well as loss of cervical lordosis [9]. Contrast enhancement of the enlarged epidural space can be observed. The initial progression of this disease usually reaches a stagnation at some point in its course.

The patient’s age as well as the lack of aggravation related to cervical flexion does not support this diagnosis.

## Histology and Immunohistochemistry

**Fig. 3:**
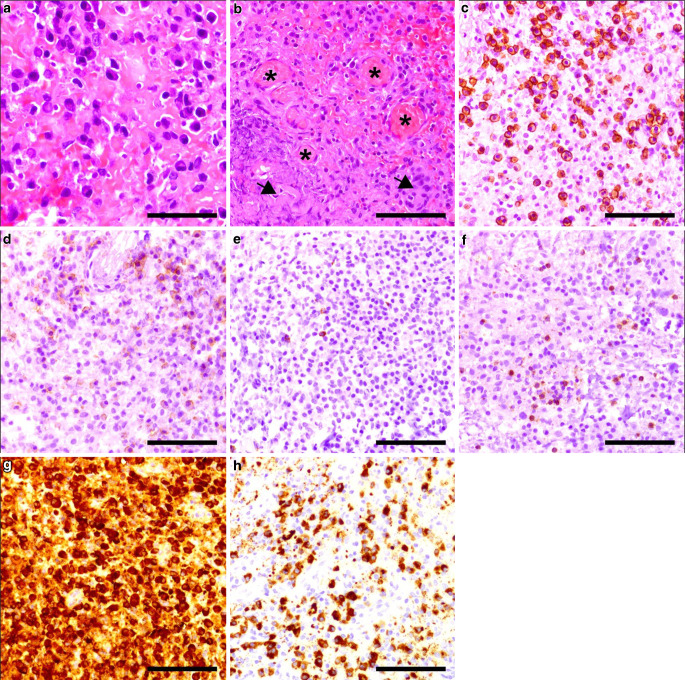
Histological examination of the lesion. **a** H&E staining showed mixed cell infiltrates. The scale bar represents 100 µm. **b** H&E staining showing obliterated blood vessels (*arrows*) with associated activation of endothelial cells and (*asterisk*) showing neovascularization. The scale bar represents 100 µm. **c** The characteristic cells showed strong cell-membrane associated CD138 positivity. The scale bar represents 100 µm. **d** CD38 immunohistochemistry was weakly positive. The scale bar represents 100 µm. **e** CD20 and **f** CD3 immunohistochemistry showed singular B and T cells, respectively. The scale bar represents 100 µm. **g** IgG immunohistochemistry was diffusely positive. **h** IgG4 was positive in individual cells. The scale bar represents 100 µm

Hematoxylin and eosin (H&E) staining of the biopsy samples showed extended mixed lymphoplasmacytic infiltrates with predominantly oval-shaped cells with eccentric nuclei and eosinophilic cytoplasm, typical for plasma cells (Fig. 3a). The tissue surrounding the lymphoplasmacytic infiltrates appeared eosinophilic (Fig. 3a). Pathogen material, accumulations of granulocytes, granuloma formation, Langerhans cells or residuals of chronic hemorrhages were not observed. Likewise absent were hallmarks of malignancy, such as mitotic spindles or tumor necrosis. Obliterated blood vessels and concomitant neovascularization were present (Fig. 3b). The predominant plasma cells showed a strong cell membrane bound immunopositivity for CD138 (Fig. 3c) and a less pronounced positivity for CD38 (Fig. 3d). CD20 (Fig. 3e) and CD3 (Fig. 3f) immunopositivity was found in single B and T cells, respectively. IgG immunohistochemistry was diffusely positive within the lesion (Fig. 3g) and IgG4 was found in approximately 40–50% of the plasma cells (Fig. 3h). Thus, the observed IgG4-positive cell density was above the diagnostically relevant cut-off of > 10 positive cells per high power field (corresponding to approximately 0.14 mm^2^) and 40% of IgG positive cells [14].

**Fig. 4:**
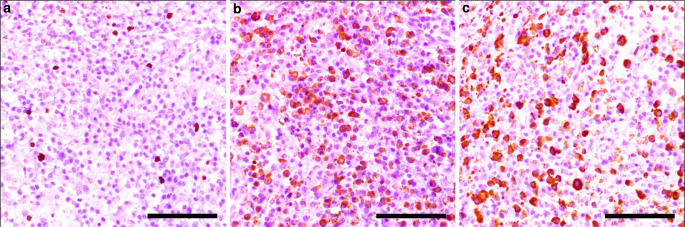
Immunohistochemical plasma cell characterization. **a** MIB-1 immunohistochemistry (**a**) revealed a positivity rate of approximately 1–2%. The scale bar represents 100 µm. The (**b**) kappa to (**c)** lambda light chain immunopositivity ratio was at a physiological level of approximately 2:1. The scale bar represents 100 µm

Histological differential diagnoses included plasma cell malignancy, vasculitis, Langerhans histiocytosis, neurosarcoidosis, tuberculous pachymeningitis, an acutely or chronically reactive process, e.g. abscess membrane or granulation tissue and an immune-mediated plasma cell-rich process. KI-67 immunohistochemistry with the monoclonal antibody clone MIB 1 revealed an attenuated proliferation rate of around 1–2% (Fig. 4a). Kappa and lambda light chain immunohistochemistry showed a kappa/lambda ratio of approximately 2:1, suggesting a non-clonal process (Fig. 4b,c). Typical changes associated with vasculitis beyond vascular obliteration were not found. Langerhans cells were absent. Granulomas, multinucleated giant cells or caseous necrosis were not observed. The lack of pathogenic material, granulocyte infiltrates and necrotic tissue argued against an abscess. Furthermore, the presence of a reactive process was unlikely due to the lack of history of surgery or trauma at the site of the lesion.

In summary, the detection of IgG4 in conjunction with a clinical history of rheumatoid pathology corroborated the clinical tentative diagnosis of a IgG4-related pachymeningitis.

## Diagnosis

### IgG4-related Spinal Pachymeningitis

IgG4-related pachymeningitis is observed in the context of IgG4-related systemic disease [15, 16]. IgG4-related systemic disease was first defined in 2003 [17]. The estimated prevalence in Japan is approximately 1 case per 100,000 population [18]. A case series of more than 100 cases has shown a male predominance and an average age at evaluation of approximately above 50 years with multiple organ systems that can be affected [19]. The organ-dependent clinical symptoms are caused by cell infiltrates, IgG4 deposition and fibrosis [15]. Histopathology along with clinicopathological assessment is the gold standard for diagnosis of this disease [20]. Typical histological features are lymphoplasmacytic cell infiltrates, obliterative phlebitis and fibrosis [20]. Ten or more IgG4-positive plasma cells per high power field depending on the examined organ confirm the diagnosis of IgG4-related disease [20].

IgG4-related systemic disease is the most common cause for hypertrophic pachymeningitis [2]. Other central nervous system manifestations include hypophysitis and, rarely, lesions of the brain parenchyma [2]. Sinus, orbital and mastoid involvement has been reported [21–23]. The treatment of IgG4-related systemic disease includes steroids, steroid-sparing agents, such as azathioprine, and B cell depletion with biologicals, such as rituximab [15]. The initial therapeutic response to steroids constitutes a relief of immunological symptoms and is seen in a majority of patients [19, 24]; however, response may vary with the degree of fibrosis in advanced disease [15]. Pachymeningitis showed a response to steroid treatment in different independent studies [25]. Therapy duration depends on various factors including the extent of the disease [26]. Compressive and obstructive symptoms due to fibrosis may require surgical debulking [26]. The prognosis is limited by disease relapses in a majority of the patients and organ specific complications that include, among others neurological and metabolic complications, aortic aneurysms and potentially malignancies [26]. In summary, IgG4-related pachymeningitis is a relatively rare disorder that may occur alongside other organ manifestations of IgG4-related systemic disease and requires an interdisciplinary approach for diagnostics and clinical management.
